# Initial analysis of the dosimetric benefit and clinical resource cost of CBCT‐based online adaptive radiotherapy for patients with cancers of the cervix or rectum

**DOI:** 10.1002/acm2.13425

**Published:** 2021-09-16

**Authors:** Adam D. Yock, Mahmoud Ahmed, Diandra Ayala‐Peacock, A. Bapsi Chakravarthy, Michael Price

**Affiliations:** ^1^ Department of Radiation Oncology Vanderbilt University Medical Center Nashville Tennessee USA; ^2^ Department of Radiation Oncology Duke University Medical Center Durham North Carolina USA; ^3^ Department of Radiation Oncology Columbia University Medical Center New York New York USA

**Keywords:** adaptive radiotherapy, adaptive replanning, adaptive therapy, ART, motion management, online adaptive radiotherapy

## Abstract

**Purpose:**

This provides a benchmark of dosimetric benefit and clinical cost of cone‐beam CT‐based online adaptive radiotherapy (ART) technology for cervical and rectal cancer patients.

**Methods:**

An emulator of a CBCT‐based online ART system was used to simulate more than 300 treatments for 13 cervical and 15 rectal cancer patients. CBCT images were used to generate adaptive replans. To measure clinical resource cost, the six phases of the workflow were timed. To evaluate the dosimetric benefit, changes in dosimetric values were assessed. These included minimum dose (Dmin) and volume receiving 95% of prescription (V95%) for the planning target volume (PTV) and the clinical target volume (CTV), and maximum 2 cc's (D2cc) of the bladder, bowel, rectum, and sigmoid colon.

**Results:**

The average duration of the workflow was 24.4 and 9.2 min for cervical and rectal cancer patients, respectively. A large proportion of time was dedicated to editing target contours (13.1 and 2.7 min, respectively). For cervical cancer patients, the replan changed the Dmin to the PTVs and CTVs for each fraction 0.25 and 0.25 Gy, respectively. The replan changed the V95% by 9.2 and 7.9%. The D2cc to the bladder, bowel, rectum, and sigmoid colon for each fraction changed −0.02, −0.08, −0.07, and −0.04 Gy, respectively. For rectal cancer patients, the replan changed the Dmin to the PTVs and CTVs for each fraction of 0.20 and 0.24 Gy, respectively. The replan changed the V95% by 4.1 and 1.5%. The D2cc to the bladder and bowel for each fraction changed 0.02 and −0.02 Gy, respectively.

**Conclusions:**

Dosimetric benefits can be achieved with CBCT‐based online ART that is amenable to conventional appointment slots. The clinical significance of these benefits remains to be determined. Managing contours was the primary factor affecting the total duration and is imperative for safe and effective adaptive radiotherapy.

## 1 INTRODUCTION

In radiotherapy, daily variations in patient positioning and anatomic motion cause the dose distribution delivered to the patient to differ from that in the original treatment plan. This difference can negatively affect the therapeutic benefit of the treatment by compromising tumor control and/or increasing the risk of normal tissue toxicity. Adaptive radiotherapy (ART) was proposed by Di Yan et al. in 1997 as a method to mitigate these effects through a feedback loop in which treatment plans are modified in response to an observed signal such as changes in the patient's anatomy.^1^ Adapting treatment plan parameters to the patient's changing anatomy allows clinicians to better achieve the original dosimetric goals.

Over the last few decades, ART has been implemented in a variety of ways to a number of different treatment sites. In head and neck radiotherapy, changes in patient anatomy that trend over the course of treatment, such as a decrease in tumor volume and a medial shift of the parotid glands, are well‐documented.^2^, ^3^, ^4^, ^5^ Offline ART—whereby the treatment planning process is repeated one or more times during a course of radiotherapy, occurring in between treatment fractions—allows clinicians to account for these types of changes. An important characteristic of this offline replanning effort is that it can be achieved by using conventional treatment planning systems and processes. The standard treatment planning workflow can simply be repeated based upon the latest representation of the patient's anatomy. This additional offline replanning effort has been found to provide a dosimetric benefit to head and neck cancer patients with trending anatomic changes.^5^, ^6^, ^7^, ^8^, ^9^ However, this improvement exhibits a diminishing return with the continued addition of successive offline replans, prompting questions about when the additional replanning effort may no longer be clinically justified.^8^, ^10^, ^11^, ^12^

In contrast to offline ART, online ART—whereby a treatment plan is recreated based on daily imaging of the patient currently on the treatment machine—represents a very different scenario with respect to the required clinical resources and the resulting dosimetric effect. Online ART can account for random variations in patient positioning and anatomic motion specific to a particular treatment fraction in addition to the systematic and trending anatomic changes amenable to offline ART. In some treatment sites, random, interfractional variations can be large, and may constitute the primary concern regarding the reproducibility of the patient's anatomy. A common example is the effect of day‐to‐day variation in the bladder and rectal filling observed in patients treated with pelvic radiotherapy.^13^, ^14^, ^15^, ^16^, ^17^, ^18^, ^19^, ^20^ Addressing these random variations requires the ability to quickly perform all of the replanning tasks at the time of treatment with the patient already in position. Conventional treatment planning and delivery systems tend to lack this functionality. However, several technologies have emerged that facilitate online ART by making imaging and treatment planning functionality available right at the treatment console. Conventionally, tasks included in the treatment planning process take a considerable amount of time. To mitigate the time associated with the replanning effort, these systems use fast computer hardware and intelligent computer algorithms to accelerate the tasks, thereby facilitating their practical use for online ART. These include systems that adapt treatment plans based on daily‐acquired MRIs^21^, ^22^, ^23^, ^24^, ^25^, ^26^, ^27^ and CBCTs.^28^, ^29^ Experience with both types of systems remains limited with their optimal implementation unknown. In the case of a new CBCT‐based system, extensive experience and guidance is largely, as of yet, unreported.

While online ART may improve the dose delivered to the patient in the presence of large, interfractional motion, its resource‐intensive nature requires careful consideration of its implementation. Treatment planning tasks performed by physicians, physicists, and dosimetrists in the weeks leading up to the start of a conventional course of treatment will now have to be accomplished in some form at the treatment console for potentially every treatment fraction. This draws a considerable amount of clinical resources to the daily treatment of an individual patient, limiting the availability of these resources for other clinical tasks. The cost in required clinical resources must be accounted for alongside the dosimetric benefit to implement online ART effectively without adversely affecting other patients or the broader clinical operation. Furthermore, the considerations of implementing an online ART workflow are complex as the experience will depend heavily on clinic‐specific factors such as the clinic size, technology, staffing level and roles, patient load and disease distribution, as well as on the local health care environment.

The purpose of this work was to provide an initial assessment of the clinical resource cost and dosimetric benefit of a new CBCT‐based online ART platform, and to characterize this experience in a way that could extend as guidance for implementation of this new technology in a variety of clinical environments.

## 2 MATERIALS AND METHODS

### 2.1 Patients

A preclinical software emulator of a CBCT‐based online ART treatment system (Ethos, Varian Medical Systems, Palo Alto, CA) was used to retrospectively simulate adaptive treatment sessions for previously treated patients diagnosed with nonmetastatic cancers of the cervix or rectum. Patients with localized cancer of the cervix or localized adenocarcinoma of the rectum for whom radiation was a component of treatment management were identified from a clinical treatment database after filtering based on ICD 9/10 diagnosis codes, treatment status, and pathological criteria to be included in this study. Target contours had been generated for cervical cancer patients using a single simulation CT of the patient with either a full or empty bladder, and for rectal cancer patients using a simulation CT acquired with the patient in the prone position. Patient contours were reviewed and standardized, adjusted by a radiation oncologist when necessary to promote consistency. Prescriptions were also standardized. Patients with cervical cancer were treated with 45 Gy in 25 fractions (1.8 Gy per fraction), and with 50 Gy given as a simultaneous integrated boost to any involved lymph nodes. Patients with rectal cancer were treated with 45 Gy in 25 fractions covering at‐risk nodal regions and 50 Gy in 25 fractions covering the gross disease.

For cervical cancer patients, the primary clinical target volume (CTVp) was contoured as the combination of the cervix, uterus, involved vagina, and parametria excluding nearby organs‐at‐risk. The primary planning target volume (PTVp) was derived by expanding the CTVp with 0.5 cm margins laterally and 1.0 cm margins in all other directions. Nodal regions to receive 45 Gy (CTVn_45 Gy) were contoured and expanded with a 0.5 cm isotropic margin to generate the PTVn_45 Gy. Any involved lymph nodes were contoured (GTVn_50 Gy) and expanded with a 0.5 cm isotropic margin while avoiding organs‐at‐risk to create the CTVn_50 Gy, which was subsequently expanded with another 0.5 cm isotropic margin to create the PTVn_50 Gy.

For rectal cancer patients, the gross disease was contoured (GTV_50 Gy) and expanding using 1.5 cm margins radially and 2.5 cm margins superiorly and inferiorly to create the CTV_50 Gy, which was subsequently expanded with a 0.5 cm isotropic margin to create the PTV_50 Gy. At‐risk nodal volumes were contoured (CTV_45 Gy) and expanded with a 0.5 cm isotropic margin to create the PTV_45 Gy.

### 2.2 Simulated treatment planning and delivery

A treatment planning template was used to standardize prescriptions, dose‐volume objectives, and objective priority weights for the two treatment sites. For each patient, multiple treatment plans were created with the following beam configurations: 7‐, 9‐, and 12‐field static gantry IMRT, and 2‐ and 3‐arc VMAT. These plans were compared and the configuration that seemed to optimally satisfy the desired dose objectives was selected.

All CBCTs acquired during the first 25 fractions of each patient's historical treatment were collected from the clinical database. For both treatment sites, CBCTs were typically acquired for roughly the first week of treatment, followed by weekly acquisition thereafter, although, in approximately one in five patients, the observed variation was large enough that daily CBCTs were acquired for the duration of the treatment course. Through remote desktop access, online ART treatments were simulated in the software emulator by using each previously acquired CBCT as a new, daily acquired, patient positioning image that would then serve as the basis for an adaptive replan. As all patients had been treated prior to the clinical availability of the described online ART system, the CBCTs had been acquired using several other treatment units (TrueBeam and Trilogy, Varian Medical Systems). Each CBCT was acquired with a protocol appropriate for the treatment site anatomy. The treatment workflow of this online ART system is composed of the following three stages: determination of influencer structure contours, determination of target contours, and treatment plan selection. Influencer structures are defined as normal tissues that, based on their positional variability and proximity to the treatment target, have significant influence over the position and distortion of the treatment target. Each of the three stages consists of an initial computational phase followed by a phase in which clinicians review, edit, and approve the results prior to proceeding to the next stage. In total, the six phases in the workflow can be described as (1) influencer structure processing, (2) influencer structure review and approval, (3) target contour processing, (4) target contour review and approval, (5) plan creation, and (6) plan review and approval. Each adaptive treatment simulated in the emulator was recorded with screen capture software to provide an independent measure of time spent throughout the workflow.

### 2.3 Timing data acquisition

This work assessed the clinical resources required for online ART by evaluating the time spent in different phases of the workflow. While clinical resources include additional considerations such as staffing levels and computing power, timing data was selected here as a broadly applicable metric. Even if different clinical environments vary in their current and potential levels of staffing and computing power, timing data is transferable and can serve as the basis for interpreting the potential effects of implementing an online ART workflow.

To acquire the timing data of the online ART workflow, screen capture videos of the simulated treatments were reviewed and time points representing the nominal transition between each of the six phases as specified in the user interface were noted. From these, the duration of each phase was determined. For each phase, the mean and standard deviation of the phase duration was calculated across all fractions for each individual patient. Subsequently, the mean and standard deviation of these patient‐specific means and patient‐specific standard deviations were calculated across the population of patients. The population mean of the patient‐specific mean was interpreted as the typical duration of each phase. The population standard deviation of the patient‐specific mean was interpreted as the interpatient variation of the duration of each phase. The population mean of the patient‐specific standard deviation was interpreted as the typical intrapatient variation of the duration of each phase. Lastly, the population standard deviation of the patient‐specific standard deviation was interpreted as the interpatient variation of the intrapatient variation of the duration of each phase.

### 2.4 Dose data acquisition

Dosimetric data in the form of dose‐volume histograms from each simulated treatment session were stored in our in‐house ART database. This included data resulting from the original plan as well as the adaptive replan, both having been (re)calculated on the anatomy depicted in that treatment's CBCT. The online ART system uses deformable image registration between the simulation CT and the daily CBCT to create a synthetic CT with accurate Hounsfield numbers and sufficient field‐of‐view to be used for dose calculation and adaptive replan optimization. The values of several dosimetric objectives were compared between the original plan and the adaptive replan. For target structures, such as the planning target volumes (PTVs) and clinical target volumes (CTVs), these dosimetric values included the minimum dose (Dmin) and the volume of the structure receiving 95% of the prescription dose (V95%). For organs‐at‐risk, such as the bladder, bowel, rectum, and sigmoid colon, these values included the maximum dose to 2 cc's (D2cc) of the structure representing the appreciable maximum dose. A two‐tailed, paired student's *t*‐test was used to compare the dosimetric values between the two plans for each structure individually (significant at *p* < 0.05).

## 3 RESULTS

### 3.1 Timing data analysis

**FIGURE 1:**
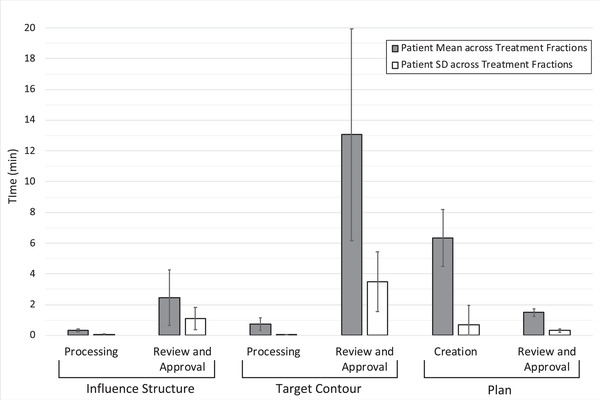
Duration of the six phases of the online ART workflow for cervical cancer patients. The height of the grey bars depicts the population average of patient‐specific duration averages, representing the typical duration of each phase. The height of the white bars depicts the population average of patient‐specific duration standard deviations, representing typical variation across treatment fractions for an individual patient. Error bars represent ±1 standard deviation calculated across the population of patients, representing the interpatient variation of these measures

**FIGURE 2:**
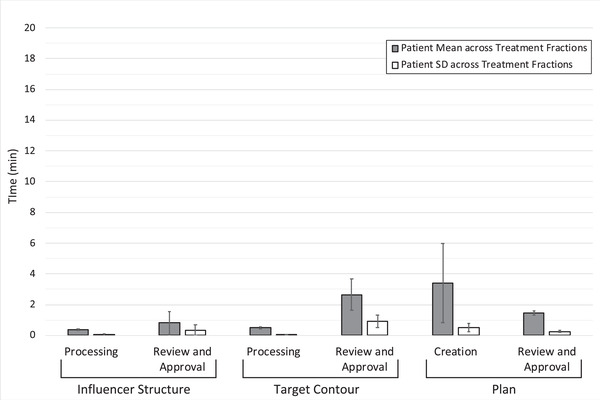
Duration of the six phases of the online ART workflow for rectal cancer patients. The height of the grey bars depicts the population average of patient‐specific duration averages, representing the typical duration of each phase. The height of the white bars depicts the population average of patient‐specific duration standard deviations, representing typical variation across treatment fractions for an individual patient. Error bars represent ±1 standard deviation calculated across the population of patients, representing the interpatient variation of these measures

Online ART treatment fractions were simulated for 13 patients with cervical cancer (including two patients with status posthysterectomy) (149 total adaptive fractions) and 15 patients with rectal cancer (162 total adaptive fractions). The overall duration of the simulated ART workflow was 24.4 min (SD = 6.9) and 9.2 min (SD = 3.2) for the cervical cancer and rectal cancer patients, respectively. Statistics regarding the individual duration of the six phases that compose the online ART workflow are depicted in Figure 1 for cervical cancer patients and in Figure 2 for rectal cancer patients.

### 3.2 Dose data analysis

**FIGURE 3:**
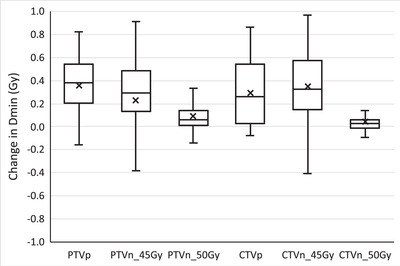
Change in the minimum dose (Dmin) to cervical cancer patient target structures for each treatment fraction when selecting the online adaptive replan. PTVp, primary planning target volume; PTVn, nodal planning target volume; CTVp, primary clinical target volume; CTVn, nodal clinical target volume. Digits in target names refer to prescription doses in gray. Any outliers are excluded from the figure

**FIGURE 4:**
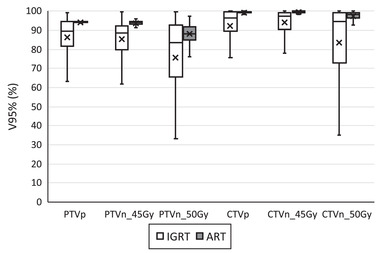
Volume of cervical cancer patient target structures receiving 95% of the prescription dose (V95%) for each treatment fraction from the online adaptive replan (ART) and the original plan recalculated on CBCT anatomy (IGRT). PTVp, primary planning target volume; PTVn, nodal planning target volume; CTVp, primary clinical target volume; CTVn, nodal clinical target volume. Digits in target names refer to prescription doses in gray. Any outliers are excluded from the figure

Figures 3 and 4 depict the differences in the Dmin and V95% for cervical cancer patient target structures between the adaptive replan and the original plan recalculated on the CBCT anatomy for individual treatment fractions. Using the adaptive replan changed the Dmin to the PTVs and CTVs for each fraction on an average of 0.25 Gy (SD = 0.33) and 0.25 Gy (SD = 0.30), respectively. The replan also changed the V95% to these target structures on an average of 9.2% (SD = 14.3) and 7.9% (SD = 13.8). The differences in both the Dmin and the V95% between the two plans for each individual target structure were statistically significant with *p* ≤ 0.001.

**FIGURE 5:**
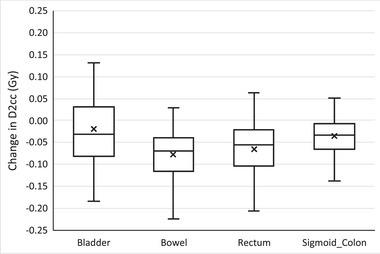
Change in the maximum dose to 2 cc's (D2cc) of organs‐at‐risk for each treatment fraction when selecting the online adaptive replan for cervical cancer patients. Any outliers are excluded from the figure

Figure 5 depicts the decrease in the D2cc of the organs‐at‐risk resulting from the adaptive replan for cervical cancer patients. The D2cc to the bladder, bowel, rectum, and sigmoid colon for each fraction changed an average of −0.02 Gy (SD = 0.09), −0.08 Gy (SD = 0.06), −0.07 Gy (SD = 0.07), and −0.04 Gy (SD = 0.05), respectively. The differences in the D2cc were statistically significant for all organs‐at‐risk with *p* = 0.017 for the bladder and *p* < 0.001 for all others.

**FIGURE 6:**
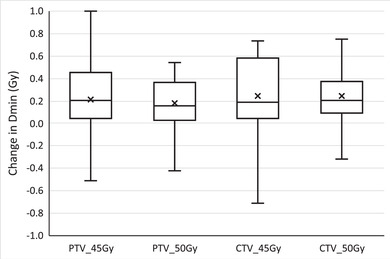
Change in the minimum dose (Dmin) to rectal cancer patient target structures for each treatment fraction when selecting the online adaptive replan. PTV, planning target volume; CTV, clinical target volume. Digits in target names refer to prescription doses in gray. Any outliers are excluded from the figure

**FIGURE 7:**
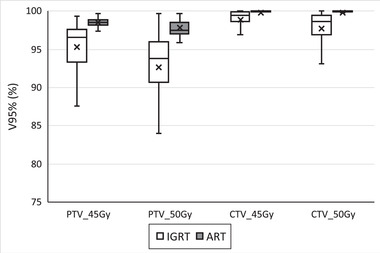
Volume of rectal cancer patient target structures receiving 95% of the prescription dose (V95%) for each treatment fraction from the online adaptive replan (ART) and the original plan recalculated on CBCT anatomy (IGRT). PTV, primary planning target volume. Digits in target names refer to prescription doses in gray. Any outliers are excluded from the figure

**FIGURE 8:**
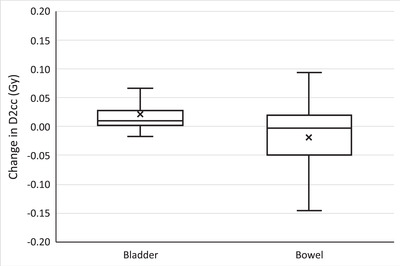
Change in the maximum dose to 2cc's (D2cc) of the bowel and bladder for each treatment fraction when selecting the online adaptive replan for rectal cancer patients. Any outliers are excluded from the figure

A similar analysis is presented for the patients with rectal cancer in Figures 6, 7, 8. Figures 6 and 7 show the differences in target structure Dmin and V95% from the adaptive replan and the original plan recalculated on the CBCT anatomy. Using the adaptive replan changed the Dmin to the PTV and CTV for each fraction on an average of 0.20 Gy (SD = 0.25) and 0.24 Gy (SD = 0.29), respectively. The replan also changed the V95% to these target structures on an average of 4.1% (SD = 4.0) and 1.5% (SD = 2.4). The differences in both the Dmin and the V95% between the two plans for each individual target structure were all statistically significant with *p* < 0.001.

Figure 8 depicts the change in D2cc for individual fractions from the adaptive replan for patients with rectal cancer, with an average change of 0.02 Gy (SD = 0.03) and −0.02 Gy (SD = 0.08) to the bladder and bowel, respectively. The differences in the D2cc were statistically significant with *p* < 0.001 for the bladder and *p* = 0.011 for the bowel.

## 4 DISCUSSION

This work evaluated the cost and benefit of a CBCT‐based online ART workflow by measuring the duration and dosimetric effect of simulated adaptive treatments for cervical cancer and rectal cancer patients. Because this work describes the duration of the treatment workflow in terms of six individual phases, the time, and clinical resources required to perform online ART can be determined even when the tasks of a particular phase are assigned to different individuals or clinical roles. Similarly, because the benefit is presented in terms of dose difference per treatment, this work can be used to approximate the effects of online ART implementation schemas that vary in the timing and frequency of adaptive replans. This represents some of the first experience with this CBCT‐based online ART treatment system, and by considering the duration and dosimetric effects together, these results can inform clinicians on how best to implement online ART.

For both cervical cancer and rectal cancer patients, creating an adaptive replan based on the CBCT tended to improve the dosimetric parameters by increasing the coverage of target structures and decreasing the maximum dose to organs‐at‐risk. Overall the dosimetry improved, although the average change remained relatively small, and several treatment fractions exhibited worse dosimetric values for one or more of the objectives. While some of these improvements were small in magnitude, it is worth noting that they corresponded to a single treatment fraction, and a larger benefit may accumulate over the course of treatment. In addition, the exact clinical impact of small improvements in delivered dosimetry remains unclear as clinicians traditionally evaluate dose distributions based on static anatomy as depicted in pretreatment simulation images, rather than those based on CBCTs of the patient currently in the treatment position.

Several other investigators have analyzed the effects on target coverage and dose to organs‐at‐risk when implementing various adaptive procedures. Oh et al. simulated multiple ART techniques that combine bony or soft tissue MRI image guidance with and without offline adaptive replanning conducted once or on a weekly basis for patients with cervical cancer.^30^ Compared to the nominal plan, the average differences in the D2cc of the bladder, bowel, rectum, and sigmoid colon as calculated from dose accumulated using the ART techniques ranged from −0.96 to 0.10 Gy. In addition, the average differences in the V45Gy of these organs ranged from −3.9 to 6.4%.

Kerkhof et al. also conducted a planning study evaluating the dosimetric effect of ART for cervical cancer using multiple MRIs.^31^ They compared an online ART protocol using weekly MRIs and smaller target margins with a reference plan based on a single pretreatment MRI with larger margins. The difference in dose to the bladder, bowel, rectum, and sigmoid colon were evaluated at six dose levels (10, 20, 30, 40, 42.8, and 45 Gy), and statistically significant differences were observed for all comparisons except at the lowest dose level for the bladder and sigmoid colon. The changes to the V45Gy for the bladder, bowel, rectum, and sigmoid colon (approximately 23, 9, 33, and 28%, respectively) were notably larger than those reported by Oh et al., likely due in part to differences in target margins used for each treatment technique.

Lutkenhaus et al. conducted a dosimetric comparison of online ART for rectal cancer using a plan‐of‐the‐day approach.^20^ For this approach, the authors created five PTVs with varied anterior margin expansions of the upper mesorectum (−2.5, −1.5, 0, 1.5, and 2.5 cm). Of the five PTVs, the three that seemed most appropriate based on the simulation image were identified and used to generate treatment plans available for online ART. At each treatment fraction, the plan featuring the smallest PTV that covered the entire mesorectum on the CBCT was selected. Per treatment fraction, the bowel cavity V95% and V15Gy were observed to decrease 8.1 cc and 13.9 cc on average, respectively, and the bladder V95% and mean dose decreased 6.7% and 0.27 Gy on average, respectively.

While the work presented here focused on patients with cervical cancer or rectal cancer, several investigators have similarly analyzed various ART techniques for prostate cancer patients, which share several organs‐at‐risk. Qin et al. compared daily IGRT alignment with online ART replanning using CBCT images to find that online ART increased the average dose to 99% of the CTV (D99%) by 1.8 Gy and also decreased doses to several organs‐at‐risk such as the rectal wall (D5% decreased 0.9 Gy) and bladder (D1% decreased 1.6 Gy).^32^ In addition to these and several other dosimetric parameters, the equivalent uniform doses to each organ from the various techniques were compared for a more holistic dosimetric analysis.

Dunlop et al. also analyzed the dosimetric effect of online ART for prostate using an MRI‐linear accelerator.^33^ They, too, observed decreases in doses to organs‐at‐risk such as the V95% to the rectum which decreased by 0.5%. However, a 3.5% increase in the bladder V95% and a 0.8% increase in the bowel D0.01cc were observed, illustrating that the benefits of replanning may not universally afford dosimetric improvement for all organs in all scenarios, and that they depend on numerous interrelated considerations regarding the plan reoptimization.

While these analyses along with the work presented here continue to describe the potential dosimetric improvements of various ART techniques, direct comparison of results proves challenging for several reasons. First, there remains a broad variety of implementation techniques for ART. These may differ in the frequency of adaptation, the imaging used, the plan generation method, and the plan selection criteria. In addition, analyses vary in precisely which of the many related dose distributions are being compared. Comparisons may include, but are not limited to, the dose of the original plan, the dose of the original plan under image‐guided patient alignment, the dose of the original plan recalculated on daily imaging acquired at a particular frequency, or the dose of an adaptive replan generated on daily imaging acquired at a particular frequency. Each of these dose distributions may, in turn, be compared on a per‐fraction basis or as a cumulative dose representing the entire course of treatment. Furthermore, the metrics reported by these studies may differ. For example, in describing the dose to a particular organs‐at‐risk, one study might report the maximum dose to a single voxel (Dmax), while another reports the maximum dose to an appreciable volume (D2cc), and a third may not report the maximum dose at all, but instead report the volume receiving a dose near the structure's maximum value such as the prescription dose. Even when the same metric is reported, different units have been used (e.g., percent as compared to cubic centimeters).^30^, ^31^ Lastly, comparing individual metrics masks the interrelatedness of dose to different organs as reflected in the competing objectives used during plan reoptimization. Nonetheless, the work presented here is consistent with the literature in demonstrating the potential of online ART techniques to improve target coverage by several percent, and to improve organs‐at‐risk sparing by several centigray to several gray per faction. The precise values of the dosimetric effects of ART techniques will continue to come into focus as clinical experience with these systems increases.

The time required to adapt treatments for cervical cancer and rectal cancer patients using the emulator was similar to that of conventional radiotherapy appointments. Most of the simulated adaptive treatments would likely have fit into 30‐ and 15‐min treatment slots for cervical cancer and rectal cancer patients, respectively. Although the analysis presented here excludes some steps like image acquisition and beam delivery, the durations of online ART tasks that were measured are generally comparable with those of previous studies that have evaluated the time required to conduct online ART processes, even when using different online ART systems for different treatment sites.

Using an MRI‐guided system (ViewRay, Oakwood Village, OH) for abdominal SBRT, Henke et al. observed an average of 9 min (2‐24) for recontouring, 10 min (2‐24) for replanning, and 4 min (1‐14) for quality assurance.^34^ These durations are consistent with those observed by Lamb et al., also treating abdominal SBRT on a ViewRay system, where on average of 10 min (5‐22) was required for recontouring and 14 min (8‐40) was required for replanning and quality assurance.^35^ The time spent recontouring also closely matches that described by Tetar et al. on their ViewRay system (10.7 min) as well as that described by Paulson et al. using the Adapt‐to‐Position workflow on an Elekta Unity MRI‐guided system (∼11 min) (Elekta, Stockholm, Sweden).^27^, ^36^ These measurements are similar to those presented here for the Varian Ethos system, where 16.6 and 4.3 min were required to determine the influencer structure and target contours for cervical cancer and rectal cancer patients, respectively.

In addition, the time required to reoptimize the adaptive replan observed by Tetar et al. and Paulson et al., 2.9 min and approximately 5‐10 min, respectively, was similar to the experience of Winkel et al. when performing online ART for prostate (0.4‐3.6 min) and rectum (0.6‐7.8 min) treatment sites on their Elekta Unity system.^23^ Again, these measurements are similar to those presented here, where 7.8 and 4.9 min was required for plan generation and review tasks for cervical cancer and rectal cancer patients, respectively. When including the time required for data transfer and registration to the replan generation steps, the duration measured by Tetar et al. (21.2 min) and Paulson et al. (approximately 16 and 32.5 min, for Adapt‐to‐Position and Adapt‐to‐Shape, respectively) are also similar to those observed by Price et al. (average 24.9‐29.2 min) and Bohoudi et al. (12 min) on their ViewRay systems.^26^, ^37^

The most direct comparison of the measurements presented here can be made with the work of Yoon et al. who simulated adaptive treatments for head‐and‐neck cancer patients also using the Varian Ethos system.^29^ Despite simulating the process for an entirely different treatment site, the average duration required to determine the influencer structure and target contours, and to generate an adaptive replan (11.8 and 6.1 min, respectively) were in agreement with those presented here for cervical cancer and rectal cancer patients.

Overall, there is a remarkable consistency between these studies on the time required to perform similar tasks across a number of different online ART systems and different treatment sites. What should not be overlooked, however, is the variation observed in each of these measured values, illustrating that considerable variation exists on an intra‐ and interpatient basis that may exceed the differences between treatment systems and disease sites.

One treatment site‐dependent effect that is very clear is the difference in observed duration required to determine contours between cervical cancer and rectal cancer patients. The fact that the total duration of simulated adaptive treatments were, on average, 2.5 times longer for cervical cancer patients than for rectal cancer patients is due to the increased duration of finalizing the target contours. It was observed that the target contours for cervical cancer patients required considerably more edits than those for the rectal cancer patients—both in the sense that a greater number of patients required edits, and in that, a more extensive effort was required to make the corrections for those who did. The former is notable in that any edit of target contours requires the system to restart the adaptive optimization and dose calculation that had previously started at the conclusion of the Target Contour Processing phase based on the pre‐edited contours, resulting in an increased time observed during the Plan Creation phase. The latter increases the duration more directly as more extensive edits of target contours naturally require more time in the Target Contour Review and Approval phase. The comparison of the time required to adapt cervical cancer patients and rectal cancer patients emphasizes the considerable consequences of suboptimal target contours. It also underscores the need to continually improve the technology, implementation, and clinical workflow to minimize the frequency and extent of contour editing.

Figures 1 and 2 show that while the duration of each phase may vary for a particular patient across treatment fractions, the greater variation was observed between patients. This result was not surprising as many of the factors that affect the duration of these tasks are patient‐specific. For example, a patient's particular anatomy, and patterns in the interfactional variation of their anatomy, influence the duration of each phase. Even a patient's individual tendency to follow treatment preparation instructions will influence the anatomic variability observed fraction‐by‐fraction. The relative position of the target and organs‐at‐risk, of contours derived from these structures, and of changes therein, will directly affect the degree to which a replan must adapt. Larger changes in anatomy can pose challenges for the deformable image registration and contour propagation required for the adaptive process. If the resulting contours are suboptimal, clinicians will be required to spend additional time and effort editing the target contours, extending the duration of review and approval phases of both influencer structures and target contours, as well as the duration of the Plan Creation phase, as described above.

In addition, patient‐specific considerations can also have a large effect on CBCT image quality. High‐quality CBCT imaging is imperative for accurate deformable image registration and contour propagation. Artifacts in the image can require clinicians to spend time editing contours even if the underlying change in anatomy did not pose such a challenge.

Of note is that the Plan Review and Approval phase remains relatively quick for both cervical cancer and rectal cancer patients. Ultimately, this is not unexpected and further underscores the critical importance of accurate contours. The difference in the original plan and the adaptive replan, fundamentally, is a reflection of the difference between the original set of contours and those created on the daily CBCT. If a considerable difference exists between these two sets of contours, the adaptive replan will almost certainly reflect a dosimetric improvement since it was created based on the contours by which its dosimetry is also being evaluated. In contrast, the original plan was created based on a set of contours that are different, albeit similar, to those by which its dosimetry is being evaluated. A dosimetric deficiency, and apparent inferiority of the original plan compared to the adaptive replan, is therefore, not unexpected. As a result, the time required to compare the dose distributions, dose‐volume histograms, and dose objectives of the original plan and the adaptive replan is relatively short.

An important caveat is that this can be the case whether or not the contours are actually accurate. An adaptive replan based on inaccurate contours will still likely appear to more successfully achieve the dosimetric objectives per those inaccurate contours, even when the resulting plan is not actually appropriate for the patient's current anatomy. This implies that while Plan Review and Approval can appear straightforward, it depends critically on contour integrity. Inappropriate contours can mislead clinicians during their selection between the original plan and the adaptive replan, especially when under the added time pressures of online ART.

When selecting a treatment plan, the clinical impact of the dosimetric difference between the original plan and the adaptive replan is a separate matter to be left to the discretion of the clinicians. If deemed clinically acceptable, some reasons to select the original plan even if it were to appear dosimetrically inferior to the adaptive replan, include greater familiarity with the original plan, and the opportunity for a more thorough assessment of the plan offline including with the completion of pretreatment plan‐specific QA.

Insight into the typical duration of each of the six phases in the adaptive workflow can also be used to inform clinicians as to the requirements of the clinical implementation of online ART. For each treatment fraction, if a particular individual is required for a particular task, the amount of time that individual is required to be at the treatment console can be estimated from the provided data. If an individual is responsible for multiple tasks, he/she may be required to be at the treatment console greater than the sum of these tasks, as the tasks may not occur in contiguous phases. For example, if a physician is responsible for Target Contour Review and Approval and also for Plan Review and Approval, they are likely to be present for the intermediate Plan Creation phase as well. This would represent roughly 86 and 82% of the duration of simulated cervical cancer and rectal cancer treatments, respectively. As a result, even if an individual is only responsible for one or a few tasks throughout the adaptive process, their presence may be required for the vast majority of the treatment. Furthermore, an individual might spend additional time at the treatment console if they were to arrive prior to being needed for a task. Conversely, if that individual was not available when needed for a task, the wait time is added to the duration of the adaptive process.

Lastly, it is critical to recognize that, in this current system, the cost incurred in the duration of the adaptive process is not determined by whether or not the adapted replan is selected for treatment, but by whether or not online ART is being considered for this patient at all. Selecting the original plan for treatment after comparing it with the adaptive replan provides no real‐time savings as the time‐cost of creating the adaptive replan has already been incurred. It may seem that if, for a particular treatment fraction, it was known from the onset that the original plan would be selected then the time spent performing tasks for the adaptive process could be avoided. However, in the current system, for a patient considered for online ART in any treatment fraction, the Influencer Structure Processing, Target Contour Processing, and Plan Creation phases cannot be avoided. Furthermore, because the target contours are used for automatic image‐guided alignment of the original plan, the care and attention required to ensure accurate contours remain critical.

This work describes the cost and benefit of a CT‐based online ART treatment system so as to inform how others may consider implementing the online ART workflow. However, there are a number of limitations that must be acknowledged for the data to be correctly interpreted. First, this work was conducted with a software emulator of the clinical system and not the clinical system itself. Although the workflow and functionality of the emulator resembled that of the clinical system to a high degree, it excluded the processes of CBCT acquisition, secondary dose calculation (plan‐specific quality assurance), and treatment delivery. The durations of these processes are, therefore, not included in the reported duration of the overall workflow. Also, because the emulator did not run on the same computer hardware as the clinical system, the duration of processing phases driven by the performance of that hardware may differ, and the duration of review and approval phases may have been affected by the fact that the emulator was accessed via remote desktop.

Another important consideration is that this work used historical CBCTs collected from patients treated on different treatment platforms (TrueBeam and Trilogy, Varian Medical Systems). As a result, the image quality may differ from the clinical online ART system, in particular, when using advanced reconstruction techniques like iterative CBCT. As discussed previously, image quality can have a considerable effect on the deformable image registration and contour propagation, which, in turn, can heavily influencing the time and effort required to achieve accurate contours.

A final consideration in interpreting the cost and benefit of online ART as reflected in this work is the potential for significant interuser variation. The magnitude of interobserver variations in contouring can be considerable, contributing to the overall uncertainty of the treatment planning process.^38^ Clinicians will vary in the speed and nature of their edits to influencer structure and target contours, and variations in these contours will affect the resulting adaptive replan and dose distribution.

Even with the aforementioned limitations, the data presented here can provide a valuable resource. Although the experiences of individual clinics will vary, this work simultaneously provides a previously unavailable assessment of both the clinical resource cost and the dosimetric benefit of a CBCT‐based online ART treatment system. The observations made during these simulated adaptive treatments can be used to inform decisions when considering the clinical implementation of an online ART workflow. These results can be used to anticipate the clinical resources required to achieve estimated levels of dosimetric improvement, or, inversely, to anticipate the dosimetric improvement from available clinical resources. While the clinical resources required for online ART include additional considerations such as the current and potential levels of staffing and computing power, timing data is a broadly applicable metric that is more transferable as it can more readily be “scaled” or “translated” as appropriate for different situations. With the duration of the adaptive workflow resolved into six phases, and with the dosimetric changes presented on a per treatment fraction basis, the utility of this early experience remains even when applied to different clinical environments. With the duration of each phase presented individually, the results can apply across variations in departmental resource allocation policies, and across different healthcare systems where working group job descriptions and responsibilities may be different.

While this work provides previously unavailable reference data and experience with a new CBCT‐based online ART system, future work reflecting actual clinical experience with this system will provide additional impact. Using similar methods with the clinical system will address a number of the limitations of this work, and provide additional insight into the function of the system within an actual clinical environment. These methods also provide a structure for more complex analyses pertaining to variations in structure contours and in dosimetric values. Other future work will benefit from comparing the experiences across multiple clinic sites and from a more detailed analysis of variations observed between patients and between users. In addition, understanding the full implications of the dosimetric effects of online ART on clinical outcomes is critical information that remains to be explored in future work as online ART becomes more prevalent.

## 5 CONCLUSIONS

In conclusion, simulations showed that adaptive replans created using a CBCT‐based, online ART system improved target coverage and decreased the maximum dose to nearby organs‐at‐risk to a statistically significant extent. However, the dosimetric benefits observed per fraction were relatively small and their cumulative effects over a full course of treatment and the corresponding clinical significance are still to be determined. The duration of the online ART workflow varied considerably between patients and between disease sites. In most cases, the duration of the workflow was similar to that of many conventional radiotherapy appointments. However, the need to perform treatment planning tasks during each treatment requires additional consideration regarding staffing and workflow. The longest portion of the process was that spent reviewing and editing target contours, and improved contour determination represents one of the most significant opportunities to decrease the resource burden of online ART. Avoiding an excessive demand on clinical resources is critical for implementing online ART techniques without disrupting broader clinical operations.

## CONFLICT OF INTEREST

AY has received consultation compensation and research support from Varian Medical Systems.
